# A Comprehensive *in vitro* and *in silico* Analysis of Nematicidal Action of Essential Oils

**DOI:** 10.3389/fpls.2020.614143

**Published:** 2021-01-08

**Authors:** Aditi Kundu, Anirban Dutta, Abhishek Mandal, Lalit Negi, Monika Malik, Rajshekhar Puramchatwad, Jyoti Antil, Anupama Singh, Uma Rao, Supradip Saha, Rajesh Kumar, Neeraj Patanjali, Suman Manna, Anil Kumar, Sukanta Dash, P. K. Singh

**Affiliations:** ^1^Division of Agricultural Chemicals, ICAR-Indian Agricultural Research Institute, New Delhi, India; ^2^Division of Nematology, ICAR-Indian Agricultural Research Institute, New Delhi, India; ^3^Division of Design of Experiments, ICAR-Indian Agricultural Statistical Research Institute, New Delhi, India

**Keywords:** volatile oils, gas chromatography-mass spectrometry analysis, *Meloidogyne incognita*, molecular docking, odorant response gene 1

## Abstract

Nematicidal potential of essential oils (EOs) has been widely reported. Terpenoids present in most of the essential oils have been reported responsible for their bioactivity though very less is known about their modes of action. In the present study, an *in vitro* screening of nine Eos, namely, *Citrus sinensis* (OEO), *Myrtus communis* (MTEO), *Eucalyptus citriodora* (CEO), *Melaleuca alternifolia* (TEO), *Acorus calamus* (AEO), *Commiphora myrrha* (MREO), *Cymbopogon nardus* (CNEO), *Artemisia absinthium* (WEO), and *Pogostemon cablin* (PEO) against *Meloidogyne incognita* revealed OEO, CNEO, and TEO as most effective with LC_50_ 39.37, 43.22, and 76.28 μg ml^–1^ respectively. EOs had varying compositions of mono- and sesquiterpenes determined by gas chromatography-mass spectrometry (GC-MS) analysis. The *in silico* molecular interactions screening of major EO constituents and the seven selected target proteins of the nematode indicated highest binding affinity of geraniol-ODR1 (odorant response gene 1) complex (ΔG = -36.9 kcal mol^–1^), due to extensive H-bonding, hydrophobic and π-alkyl interactions. The relative binding affinity followed the order: geraniol-ODR1 > β-terpineol-ODR1 > citronellal-ODR1 > *l*-limonene-ODR1 > γ-terpinene-ODR1. Taken together, the cumulative *in vitro* and computational bioefficacy analysis related to the chemoprofiles of EOs provides useful leads on harnessing the potential of EOs as bionematicides. The insight on biochemical ligand–target protein interactions described in the present work will be helpful in logical selection of biomolecules and essential oils for development of practically viable bionematicidal products.

## Introduction

Root knot nematodes, pose a major challenge to the global pest management programs due to devastating crop losses caused by these organisms (Sidhu et al., 2017). Among the root knot nematodes, *Meloidogyne incognita* is most abundant in tropical soils, barely sparing any crop family, and the most challenging part is to control its population below economic damage levels (Collange et al., 2011; de Freitas Silva et al., 2020). Synthetic recommended nematicides like carbofuran, fluopyram owing to their associated detrimental effects on the environment, non-target organisms besides phytotoxicity, necessitate safer approaches for nematode management in cropping systems (Westphal, 2011; Jones et al., 2017). To develop ecofriendly alternatives, a wide spectrum of plant metabolites with nematostatic and nematicidal actions has extensively been reported (Atolani and Fabiyi, 2020).

Phytochemicals have been extensively reported as potential sources of bioactive ingredients for the development of natural nematicides (Oka, 2001). Long-chain hydrocarbons, sulfur compounds, alkenes, furans, acetogenins, phenolics, saponins, etc., have been reported to be effective against various phytoparasitic nematodes (Aissani et al., 2018). Most of the phytochemicals have served as models for the identification of a lead molecule with potential commercial applications (Cantrell et al., 2012). Volatile organic compounds of botanical origin, most commonly found in essential oils, have particularly been recognized as highly effective against *M. incognita* (Aissani et al., 2015; Silva et al., 2018; Pedroso et al., 2019).

Plant essential oils (EOs) are complex mixtures of terpenoids and their oxygenated derivatives, produced by isoprenoid pathways (Tetali, 2019). Only ∼10% of the reported plant species produce EOs (Kalemba and Kunicka, 2003). Stored in secretory glands in epidermic cells, secretory hair, glandular trichomes, EOs play a key role in plant defense against biotic stresses (Bakkali et al., 2008). Known for their bioactive potential against diverse agriculturally important pests, EOs in numerous reports have been mentioned as very effective against *M. incognita* (Caboni et al., 2013). EOs of *Acorus calamus* and *Pogostemon cablin* have been tested for nematicidal activities (Perrett and Whitfield, 1995; Lee et al., 2009). EO from *Eucalyptus citriodora* was found highly toxic to *M. incognita* at 500 μl ml^–1^ by Pandey et al. (2000). *Citrus sinensis* EO was reported effective against the phytonematodes, *M. incognita*, *Pratylenchus vulnus*, and *Xiphinema index* (Avato et al., 2017). Similarly, EO of *Myrtus communis* was reported to kill 100% of *M. incognita* juveniles at 4,000 μl ml^–1^ concentration (Ardakani et al., 2013). Another study reported that EO of *Melaleuca alternifolia* was highly active against the larvae of *Anisakis simplex* at a concentration of 7 μl mL^–1^ (Andrés et al., 2012). The EO of *Cymbopogon nardus* tested against *M. incognita* exhibited moderate effectiveness in a study reported by Sinha et al. (2006). Similarly, the toxicity of *Artemisia absinthium* EO has been documented against *M. incognita* on the tomato plant (Amora et al., 2017). Promising nematicidal action of sesquiterpenes rich in the EO of *Commiphora myrrha* against juveniles of *M. incognita* was reported by Kong et al. (2006) and Ardakani et al. (2013).

A review of literature clearly showed that EO bioactivity evaluation against nematodes largely remained restricted so far to the evaluation of EOs against different plant parasitic nematodes. Emphasis on the correlation of their anti-nemic activity with chemical compositions and mechanism of interaction at molecular level with the possible target sites of action has remained lacking. Therefore, the present study was performed to characterize the chemical composition of the selected EOs, evaluate their bio-efficacy *in vitro* against *M. incognita*, and subject the most effective EOs to *in silico* analysis for a likely mode of action, using molecular docking and modeling approach.

## Materials and Methods

### Essential Oils

Commercially available EOs (99% purity) of different plants, namely, OEO (*Citrus sinensis* (L.) Osbeck; family Rutaceae, orange essential oil), MTEO (*Myrtus communis* L.; family Myrtaceae, myrtle essential oil), CEO (*Eucalyptus citriodora* L.; family Myrtaceae, citriodora essential oil), TEO (*Melaleuca alternifolia* L.; family Myrtaceae, tea tree oil), AEO (*Acorus calamus* L.; family Acoraceae, calamus essential oil), MREO [*Commiphora myrrha* (Nees) Engl; family Burseraceae, myrrh essential oil], CNEO (*Cymbopogon nardus* L. Rendle.; family Poaceae, citronella oil), WEO (*Artemisia absinthium* L.; family Asteraceae, wormwood essential oil), and PEO [*Pogostemon cablin* (Blanco) Benth.; family Lamiaceae, patchouli essential oil] were purchased from CDH Fine Chemicals (New Delhi, India) and Merck^®^, (New Delhi, India) and used without further purification.

### Chemicals and Reagents

All the solvents used were of AR grade, purchased from Merck^®^, (New Delhi, India). Surfactants, Atlas G5002 and Triton X-100 were procured from Croda India Company Pvt. Ltd. (Navi Mumbai, India) and Loba Chemie Pvt., Ltd. (Mumbai, India), respectively. For GC-MS analysis, helium (He) gas of high purity (99%) was used.

### Gas Chromatography-Mass Spectrometry Analysis

Volatile constituents of EOs were analyzed in GC-MS in a 7890A GC instrument (Agilent Technologies^®^, United States) equipped with an HP-5MS column (30 m × 0.25 μm;/0.25 μm, Agilent Co., United States) as stationary phase, which was directly connected to a triple axis HED-EM 5975C mass spectrometer (Agilent Co., United States). The injection volume was 1 μl with flow mode in split control. Helium was used as carrier gas at a head pressure of 10 psi, and flow was set at 1 ml min^–1^. The GC-MS condition was programmed with the oven temperature initially held at 40°C for 1 min, thereafter increased with a gradient of 3°C min^–1^, until the temperature reached to 120°C and held constant for 2 min. The temperature was raised again with a gradient of 5°C min^–1^ up to 220°C and held constant for 1 min and finally raised to 280°C with an increment of 4°C min^–1^. The total run time of the analysis was 65 min. The MS acquisition parameters were ion source temperature 180°C, electron ionization 70 eV, full scan mode (50–550 AMU), transfer line temperature 280°C, solvent delay 3 min, and E.M voltage 1,380 V. Compounds were identified by matching their mass spectra and fragmentation pattern using NIST (National Institute of Standards and Technologies) Mass Spectra Library. Further rentention indices (RI) have been calculated following Kovats (1978):

RI = 100 ^∗^ n + [log(RT_*compound*_ - v) - log(RT - v)]/[log(RT_*larger alkane*_ - v) - log(RT_*smaller alkane*_ - v)]

where n = the number of C in the smaller alkane, RT_*compound*_ = the retention time of the compound, v = the column void time, RT_*larger alkane*_ = the retention time of the larger alkane, and RT_*smaller alkane*_ = the retention time of the smaller alkane.

### Nematicidal Assay

#### Collection of Nematodes

Nematode culture was maintained on infected tomato plants (var. Pusa Ruby) under greenhouse conditions. Second instar juveniles (J_2_s) of *M. incognita* were collected from roots of 21-day-old infected tomato seedlings. Nematode-infested soil was screened through water screening method following Cobb’s sieving and decanting technique (Cobb, 1918). Further, nematode egg masses were picked up from the sterilized infected roots of tomato seedlings, transferred to fresh distilled water in Petri plates, and allowed to hatch under ambient condition of 27 ± 1°C for 5 days. The hatched nematode juveniles travel through soft wet tissue placed on the wire of the Petri plate on the surface water (Julio et al., 2017). Nematode J_2_s suspensions were combined and counted under light microscope.

#### Preparation of Essential Oil Emulsions

Primary stock emulsions (10,000 μg ml^–1^) of all EOs except PEO were prepared using Atlas G5002 surfactant (2% w/w). In the case of PEO, Triton X-100 (2% w/w) was used. Each primary stock emulsion was diluted serially with surfactant solution to prepare secondary test emulsions of varying strengths (1,000–10 μg ml^–1^).

#### Nematicidal Activity

Nematicidal assay was conducted under *in vitro* condition to assess the activity of the EOs against *M. incognita* following a known method with slight modifications (Kundu et al., 2016). Treatments comprised of nine Eos, namely, OEO, MTEO, CEO, TEO, AEO, MREO, CNEO, WEO, and PEO. Aqueous suspension (1 μl) containing 25 J_2_s of *M. incognita* was added to each well of multiwell plates (15.6-mm diameter), each containing EO emulsion (2 ml) of a particular test strength (1,000–10 μg ml^–1^). Surfactant solutions used to dissolve EOs were taken as corresponding negative controls. Each treatment was replicated thrice. Multiwell plates were incubated at 27 ± 1°C and examined using a stereoscopic microscope at 24, 48, and 72-h intervals. The numbers of dead vs alive juveniles in each treatment was recorded. Motionless nematodes with straight bodies were counted. The revival test was done as described by Choi et al. (2007). Briefly, the motionless nematodes were teased with a needle followed by transfer to fresh wells containing deionized water. One drop of sodium hydroxide (1M) solution was added to check any movement. Mortality (%) and corrected mortality (%) of J_2_s was calculated considering the mortality of juveniles in negative control.

### Molecular Docking and Simulation

Based on the results of *in vitro* nematicidal assay and GC-MS analysis, major volatile constituents of OEO, CNEO, and TEO were selected out of nine EOs, for *in silico* ligand target protein interaction analysis.

#### Selection of Protein

Seven target proteins, namely, cytochrome c oxidase subunit 1, AChE, Hsp90, ODR1, ODR3, neuropeptide GPCR, CLAVATA3/ESR (CLE)-related protein of *M. incognita* were selected as target receptors for the molecular docking studies. Cytochrome c oxidase subunit 1 is involved in the oxidative phosphorylation pathway, which is part of the energy metabolism. AChE regulates synaptic transmission and locomotion processes. The full functional activity of Hsp90 is gained in coordination with other co-chaperones, playing an important role in the folding of newly synthesized proteins, stabilization and refolding of denatured proteins during stress. ODR1 and ODR3 regulate chemosensory functions. Neuropeptide GPCR is associated in the regulation of movement of the parasite toward (or within) its host. CLAVATA3/ESR (CLE)-related protein plays an important role in the differentiation or division of feeding cells (syncytia) induced in plant roots during infection. (Ref. for each).

#### Protein Preparation

The hypothetical protein sequences were taken from the NCBI and UNIPROT database (Table 1). The BLAST servers^1^ ^,2^ were used to search and annotate the molecular and biological functions of the query sequences. The NCBI Blast tool and the PDB database were together used to identify the templates for modeling the secondary structures of the query sequences. Further homology modeling of the proteins was carried out using Modeller v 9.24.

**TABLE 1:** Target sequences screened for *in silico* nematicidal activity.

**Receptor**	**Amino Acid Seq**	**Source**
Cytochrome c oxidase subunit 1	LVTKSVTHKNIGFIYLFFSFWSGLMGLSLSMLLRMDLMKSGMVIGDGQLYNVILTSHALVMIFFMVMPG LIGGFGNFFFPILINCIDLFLPRVNNMSYWFLPGSLILLMFSLFMDKGSGTGWTLYPPLMI DGQPGRSTDLVIFSLHFSGISSISSGINFLSTCHEMRLEVKTLEIMSLFVWCLIITVFLLVLSLPVLASGITMGLSDRN FNTGFFDSNMGGNILMFQHLFWFFGHPEVYVLIAPAFGLVSMVMVLLSSKKDLYGRK GMILAIMSIGFIGCLVWGHHMFTVGMDHDSRAYFSSATMIIAIPTGMKIFSWMMTLYGSKLNWNYLIL WIMGFIFMFTVGGLSGLILSNAGLDIFLHDTYYVVAHFHYVLSMGAVFGIFLGFFFSYGFMFGLMMNSVLVK SFFYIFFLGVNLTFFPMHFSGLQGQPRKYMSYSSDYLFWQMFASIGS LLSLFSIFLLIYLILESMIIFRLLIFDLFSFSMVSLNVNNYFHTNLDLSMIWLK	NCBI GenBank
AChE	MRKRRRKTTAFSINTSELLRLYFKFSSHSCLTFIFCCFFCLIVYCSSVHGRSSPVALTDVLIQTTLGKIIGFKQK FDGKSVHTFLGVPYAKSPTGSGRFGLPEMIEPWEGEFRADKPARTCFFSRDTMFPDFPGAEMWNPPNDIDEDCLAMNIW VPEHHDGTVLVWIYGGGFYSGSPSLDLYDGRVLAVQERAVVININYRLGPFGFLYFGDD TSVPGNMGLQDQQMALKWIHEHIAHFGGDPRRVTLFGESAGSASAMAHMFADG SYSLFSRIIAQSGSIINNWATKPKASILQISLQLAHHLNCSNGNN STKAMQNIVECIRRVPTSIIQRAGDAVSQSLSLPMDFAFVPIDEDTHFFRGNV FDKLRRKNFKRDVSILVGTVRDEGTYWLPYCLQKNGFGFNHTISPEDHINQALISETDYTKAFDAFLPYFGN SNLVRHALMHAYSHLPTEKQEQRWRDGVARF LGDYFFTCDSIEFADIVSDELYGSVYSFYFTRRSSANPWPQWMGAMHGYEIEYVFGLPLRSPHLYDPSELELEISFSTKIMEF WGHFARTGEPVEFWPKYNRITRKSLVLSEEIATGTSHRIYVDVHGKLCRLLEEAQAVAGITGEQRSRICPDGRATTVNYGQE ISMEDVKEEMQLNRGISGINRIPSIKIYISLIILSLALLRSPEISFLYSSFIFK	NCBI GenBank
Hsp90	MSLIINTFYSNKEIFLRELISNSSDALDKIRYQALTDPAQLETGKDLYIKIVPN KADKTLTIMDTGVGMTKADLVNNLETIAKSGTKAFMEALQAGADISMIGQFGVGFYSAFLVADRVTVTSEHNDDDCHQ WESSAGGSFIIRNCVDPEMTRGTKITLYLKEDQTDYLEERRIREVVKKHSQFIGYPIKLLVEKERDKEISDDEAEDEKKDVK KEEEKEEEKEIKKEEGEDKEGEDEDKDKKDGEKKKKTKKIKEKYTE DEELNKTKPIWTRNPDDITNEEYAEFYKSLSNDWEDHLAVKHLSVEGQLEFR ALLFVPQRAPFDMFENKKQKNAIKLYVRRVFIMENCEELMPEYLNFIKGVV DSEDLPLNISREMLQQSKILKVIRKNLVKKCIELFDEIAEDKDNFKKFYEQFSKNLKLGIHEDSVNRKKLAEYL RYNTSSSGDELVSLKDYVGRMKENQTCIYYITGESKEVVQNSAFVERVKKRGFEVIYMVDPIDE YCIQQLKEFDGKKLVSVTKEGLELPESEEEKKKFEEDKVKF EKLCKVIKDILDKKVQKVSVSNRLVSSPCCIVTGEYGWTANMERIMKAQALRDSSTMG YMASKKNLEINPDHSIIKSLRERIDSDQDDKTAKDLVVLLYETALLTS GFSLEDPQQHASRIYRMVKLGLDITEEDLEGGEQQPCTSGEPVEKIAGAEEDASRMEEVD	NCBI GenBank
ODR1	MMTGQQSTESFLATLAIYNACYGFCLGSSLTSTGSFASDPNNPAFVANLRGKSFQGIKKFLLPK RNFQFKGSFGQVNLTSWPAPLQNLAIYTLPSSGGQYSLIYTAISIPSSSCGT FECFDIQLQTSPNISEDLLWQKQCSNTIPSCIYSGGCSSLVPYFSAGAAIVLVAAAAGIVYTIQRKKRLDVFRVH WRIGRQQFKVIENKQAKGKATGIGQEGAWSKRRQLHAYALIGTNKAEFIV LRQMKKIYWDKIELHFIFELKKLNHDNLTTFMGICYNDGDKFYVCHSLVERGTLEDYIHDLD FQLDNTFRSAFLRDILKGVKYLHKSSIGYHGMLNLQNVLIDSNWVLKLTNFGIGNLLNRAIRREQLQLIELIPLNTYLT VAPENLIDISYGREYPNGTTIGDIYSMGMVMYHILFRLAPYERTTLSPKEVIDQVRQHNLKPILENTLPEEK PLVDAMEQCWQKNLDLRPRLRQLAQVVSTVFQASQGNLIDQMRRMNEKHALNLEKLV TQRNAELAQAREQTERLLNEMLPPSIAAQLKEHKSV EPRSYDSATVLFCQLVDFSTVLSKFPPDQVIDFLNQVFSTFDTIIRNHDAYKVETTGETYMVAS GVPNENENRHVFEISEVAMEFREVSYTYKSINFP DWKLQLRIGYHCGPIAAGVIGIKAPRYCLFGDTVNFASRMQSNAAPNQIQMSESTALLLMGVSKYKLTKRGIVKVKGKER	WormBase ParaSite Database
ODR3	SCQSEEVREQLSKNKAIEKQLTSDRRAASSIIKLLLLGAGECGKSTVLKQMQILHSNG FTEEEINERKAVVYSNTVTSMAAILKAMDNVLHMPMDDASKERDRNLIFRAIENGEENLPFTDPIAKALQNLWGDKAVK KAYEMRSEYQLNDSAKYFLDSVSRIHEPGYRPTEQDILYSRVATTGVVEVKFIIKGNMEFRVFD VGGQRSERRKWIHCFDNVEAIIFITAISEYDQVLF EDETTNRMIESMQLFSSICNSSWFLNTAMILFLNKKDLFLEKIQRVNITTCF PDYEGSQNYEEAVNFIKMKFAELNQHPDKKTIYMHETCATDTN	WormBase ParaSite Database
Neuropeptide GPCR	MVSSISLNQQINQIEIENCIELNSVLDQFGDWTLRLDVKFFYSLFYAAIFIVGL IGNGFLVGTIRRRMTVANVFLMNLAISDLLLCITALPITPVLAFVKRWIFGLALCKLVPLCQGISVLISSY CLCLIAVDRYRSIVTPLKVPWNIQXAQWLMTLCWTFCIIISSPLFIVQGLQQIVYKNMTFCGEFCTEL NWPTDFRIKLFYGISLLSIQFLIPTLIMTYCYWKILQKVRQDWLVPTNNSIMSLEQQAQTAI RKRRVMYVLILMVLIFMGSWMPLTFVNLLRDIGISFLET QMYFKLLNVXAVAMTSVVSNPLLYFYMSKRXRRALRDDMYWLTNARRQQNQXVGGLLAKF TPSPSIGLLYKKSLERHILQNATAKYNPYRRGTLADPTTLGREKVLQEMHANCFLLVPL MPLCVANQQRLATNQREISNNNNINLNFKRQKHPKFVCEA	NCBI GenBank
CLAVATA3/ESR (CLE)-related protein	MFTNSIKNLIIYLMPLMVTLMLLSVSFVDAGKKPSGPNPGGNN	UNIPROT Database

**Meloidogyne incognita* acetyl cholinesterase (AChE), *M. incognita* heat shock protein 90 (Hsp90), *M. incognita* odorant response gene-1 (ODR1), and *M. incognita* neuropeptide G-protein coupled receptor (nGPCR).*

#### Ligand and Receptor Preparation

The molecular structures of the chemical constituents, referred hereafter as “ligands,” of OEO, CNEO, and TEO were downloaded as.sdf file from PUBCHEM database^3^. The ligand structures were minimized using MM2 forcefield in Chem Draw Ultra 11.0 software [Cambridge Soft Corp., Cambridge, MA, United States (2009)] and used for molecular modeling studies. The ligand molecules were customized for docking using the Dock prep tool of Autodock Vina. Hydrogen molecules were added, and the incomplete side chains were replaced using Dunbrack rotamer library (Dunbrack, 2006). Charges were computed using ANTECHAMBER. AMBER ff14SB and Gasteiger charges were allotted to standard residues and to other residue types, respectively. Similarly, receptor molecules were prepared using the same tools except that the ANTECHAMBER was not employed. All the prepared ligand files were saved in the Mol2 format and the receptor files in the.pdb format.

#### Molecular Docking Simulation

The customized ligand and receptor molecules were used for docking in ICM Molsoft v. 2.8.ICM software, which performed adaptable ligand docking through global optimization of the energy function (Abagyan et al., 1994). The energy functions incorporated the internal energy of the ligand in view of the ECEPP/3 drive field, and van der Waals, hydrogen-holding, electrostatic and hydrophobic ligand/receptor association terms pre-ascertained on the lattice for computational proficiency (Bursulaya et al., 2003). Flexible ligand docking with the ICM software used Monte Carlo simulations to globally optimize a set of ligand internal coordinates in the space of grid potential maps calculated for the protein pocket (Neves et al., 2012). Discovery Studio v. 4.1 Client was used to study the docked receptor–ligand interactions. The most favored docking conformation interactions of *ODR1* with geraniol, β-terpineol, citronellal, *l*-limonene, and γ-terpinene were analyzed on the basis of docking score, binding affinity, and interacting residues. The active site residues were identified, and depictions of all possible interactions in 3D and 2D poses were prepared using DS Visualiser v. 4.1.

In order to avoid affinity-based selection and optimization of larger ligands, the emphasis was given to compounds that most effectively utilized their atoms. In an attempt to measure the compound effectiveness, Hopkins et al. (2014) suggested an estimation of binding affinity of molecule, in terms of ligand efficiency (LE):

$$\text{LE}=\frac{\left[-2.303\,\left(\text{RT}\right)\,\times \log\,K\,d\right]\,}{\text{HA}}=\frac{-\Delta \,G}{\text{HA}}$$

where, ΔG is free-binding energy and HA is the number of ligand non-hydrogen atoms. LE is related to the amount and effectiveness of heavy atoms in a molecule toward complex formation. The average affinity contribution per atom was taken into consideration instead of considering the affinity of the whole compound. This enabled measuring the affinity of the corrected molecules with their size. In drug discovery modules, candidate molecules with LE values ≥ 0.3 kcal per mole per heavy atom usually are taken ahead as lead molecule (Hopkins et al., 2014).

### Statistical Analysis

The bioassay experiments were done in triplicate. The significance of the differences between variables was tested using one-way ANOVA. The means were compared using Duncan’s multiple range test. Statistical significance was determined at *p* < 0.05. Percent mortality data were subjected to probit analysis using Polo Plus software to determine lethal concentrations (LC_50_ and LC_90_, expressed in μg ml^–1^).

## Results

### Essential Oil Composition

**FIGURE 1:**
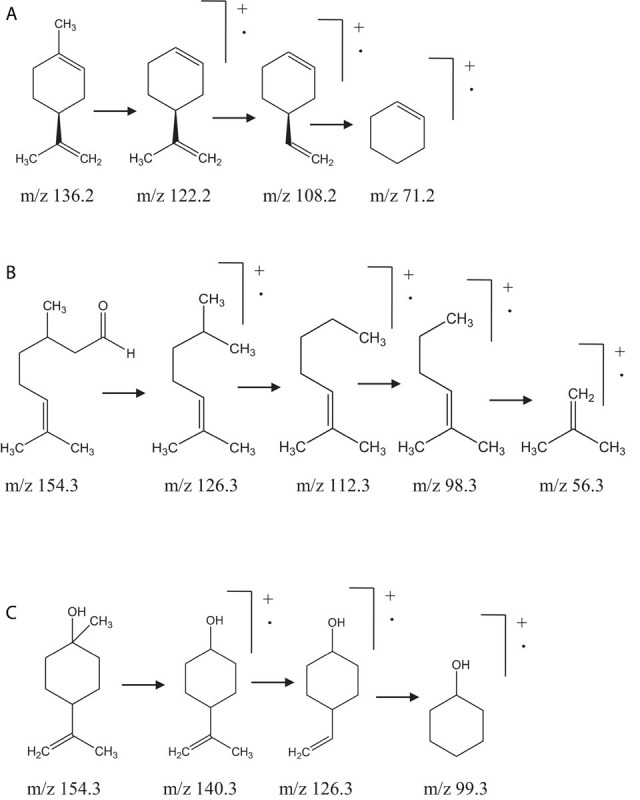
Mass fragmentation pattern of **(A)** *l*-limonene, **(B)** citronellal, **(C)** and β-terpineol.

The compositions of EOs of OEO, MTEO, CEO, TEO, AEO, MREO, CNEO, WEO, and PEO were determined by comparing their mass spectra with data library, corresponding retention indices, and mass fragmentation patterns. The identified chemical constituents of the oils are listed in Table 2. The aromatic profile of most of the EOs showed dominance of one or two major constituents. Individually, *l*-limonene (93.2 ± 2.30%) was found to be most abundant in OEO, along with β-myrcene (2.2 ± 0.2%), α-pinene (1.6 ± 0.1%), sabinene (0.5 ± 0.1%), limonene oxide (0.2 ± 0.0%), and decanal (0.2 ± 0.0%). *l*-Limonene was confirmed based on its fragmentation pattern with characteristic daughter ion peaks of m/z 136.2, 108.2, and 71.2, generated due to sequential loss of methyl and ethyl moieties (Figure 1). Interestingly, OEO was found to contain only monoterpenes. The monoterpenic constituents of MTEO were identified as α-pinene (42.3 ± 1.1%), 1,8-cineol (30.3 ± 1.3%), linalool (7.6 ± 0.9%), and linalyl acetate (6.6 ± 0.5%). GC-MS analysis of TEO showed several peaks corresponding to 27 mono and sesquiterpenoids, comprising 93.3% of the total oil. Monoterpenes (43.5%) and their oxygenated derivatives (42.6%) were found to be the most abundant. Among monoterpenes, β-terpineol (35.7 ± 1.2%) was identified as the major constituent followed by *γ*-terpinene (17.5 ± 1.1%), α-terpinene (8.7 ± 0.6%), *p*-cymene (4.8 ± 0.2%), α-pinene (3.2 ± 0.2%), *p*-menth-8-en-2-ol (3.2 ± 0.5%), and 1,8-cineol (3.1 ± 0.4%). Besides, α-gurjunene (2.1 ± 0.1%) and δ-cadinene (1.6 ± 0.2%) were identified as the major sesquiterpenes.

**TABLE 2:** Chemical composition of various EOs as analyzed in GC-MS and content (%)^*c*^ of constituents.

**Compounds^*a*^**	**RI^*b*^**	**OEO**	**MTEO**	**CEO**	**TEO**	**AEO**	**MREO**	**CNEO**	**WEO**	**PEO**
***Monoterpene hydrocarbons***										
Thujene	930	nd	0.2 ± 0.1	nd	2.3 ± 0.1	nd	nd	nd	0.1 ± 0.0	nd
*o*-Cymene	937	nd	nd	nd	nd	nd	nd	nd	5.1 ± 0.2	nd
α-Pinene	939	1.6 ± 0.1	42.3 ± 1.1	0.1 ± 0.0	3.2 ± 0.2	0.2 ± 0.1	0.1 ± 0.0	nd	2.7 ± 0.2	0.2 ± 0.0
Camphene	954	nd	4.2 ± 1.0	1.0 ± 0.1	nd	0.1 ± 0.0	0.3 ± 0.0	nd	nd	nd
*t*-Ocimene	955	0.1 ± 0.0	nd	nd	nd	nd	nd	nd	nd	nd
Sabinene	975	0.5 ± 0.1	nd	nd	nd	nd	nd	nd	5.6 ± 0.3	nd
β-Pinene	979	nd	1.0 ± 0.2	0.2 ± 0.0	1.3 ± 0.1	nd	0.2 ± 0.0	nd	0.3 ± 0.1	0.3 ± 0.0
β-Myrcene	991	2.2 ± 0.2	nd	nd	2.3 ± 0.1	0.1 ± 0.0	nd	nd	0.2 ± 0.0	nd
Phellandrene	1,003	nd	nd	nd	0.6 ± 0.0	nd	0.1 ± 0.0	nd	nd	nd
*p*-Cymene	1,013	nd	nd	nd	4.8 ± 0.2	nd	nd	0.1 ± 0.0	0.1 ± 0.0	nd
α-Terpinene	1,017	nd	nd	nd	8.7 ± 0.6	nd	nd	nd	0.9 ± 0.3	0.2 ± 0.0
*l*-Limonene	1,029	93.2 ± 2.3	nd	nd	nd	nd	nd	5.7 ± 0.5	nd	nd
δ-3-Carene	1,033	0.4 ± 0.1	1.9 ± 0.5	nd	nd	nd	nd	nd	nd	nd
β-Ocimene	1,051	nd	nd	nd	nd	nd	0.2 ± 0.0	nd	0.4 ± 0.0	nd
*γ*-Terpinene	1,060	nd	nd	nd	17.5 ± 1.1	nd	nd	nd	1.1 ± 0.2	nd
α-Terpinolene	1,089	nd	nd	nd	2.8 ± 0.3	nd	nd	nd	1.1 ± 0.2	nd
***Oxygenated monoterpenes***										
Fenchone	1,008	nd	nd	2.3 ± 0.3	nd	0.1 ± 0.0	nd	nd	nd	nd
1,8-Cineole	1,035	nd	30.3 ± 1.3	nd	3.1 ± 0.4	nd	nd	nd	10.6 ± 0.5	nd
Limonene oxide	1,087	0.2 ± 0.0	nd	nd	nd	nd	nd	nd	nd	nd
Linalool	1,089	0.4 ± 0.1	7.6 ± 0.9	nd	nd	0.2 ± 0.0	1.7 ± 0.3	0.8 ± 0.2	6.4 ± 0.4	nd
Eucalyptol	1,093	nd	nd	0.2 ± 0.0	nd	nd	nd	nd	nd	nd
*p*-Menth-8-en-2-ol	1,090	nd	nd	nd	3.2 ± 0.5	nd	nd	nd	nd	nd
*t*-Thujone	1,102	nd	nd	nd	nd	nd	nd	nd	0.3 ± 0.0	nd
Pulegol	1,116	nd	nd	nd	nd	nd	nd	0.1 ± 0.0	nd	nd
Camphor	1,146	nd	nd	nd	nd	nd	nd	nd	3.8 ± 0.5	nd
Citronellal	1,148	0.1 ± 0.0	nd	81.9 ± 1.1	nd	nd	nd	31.5 ± 1.1	nd	nd
Isopulegol	1,150	nd	nd	nd	nd	nd	nd	1.0 ± 0.1	nd	nd
β-Terpineol	1,163	nd	nd	nd	35.7 ± 1.2	nd	nd	nd	16.2 ± 0.9	nd
Borneol	1,169	nd	nd	nd	nd	0.1 ± 0.0	nd	nd	0.5 ± 0.1	nd
4-Caranol	1,185	nd	nd	nd	nd	nd	nd	nd	0.3 ± 0.0	nd
Decanal	1,202	0.2 ± 0.0	nd	nd	nd	nd	nd	nd	nd	nd
Citronellol	1,226	nd	nd	5.8 ± 0.7	nd	nd	nd	9.6 ± 0.3	nd	nd
Geraniol	1,231	nd	nd	nd	nd	nd	nd	30.6 ± 1.3	0.6 ± 0.1	nd
Citral	1,236	nd	nd	0.1 ± 0.0	nd	nd	nd	nd	nd	nd
Neral	1,240	nd	nd	nd	nd	nd	nd	0.5 ±	nd	nd
Linalyl acetate	1,257	nd	6.6 ± 0.5	nd	nd	nd	nd	nd	nd	nd
Geranial	1,270	nd	nd	nd	nd	nd	nd	0.7 ±	nd	nd
Borneol acetate	1,289	nd	nd	nd	nd	nd	nd	nd	26.6 ± 1.7	nd
Neryl acetate	1,362	nd	nd	nd	nd	nd	nd	2.1 ± 0.2	0.7 ± 0.1	nd
Geranyl acetate	1,383	nd	nd	nd	nd	0.1 ± 0.0	nd	nd	0.2 ± 0.0	nd
Thymol	1,470	nd	nd	nd	nd	nd	nd	nd	0.3 ± 0.0	nd
***Sesquiterpene hydrocarbon***										
δ-Elemene	1,343	nd	nd	nd	nd	nd	2.1 ± 0.2	nd	2.0 ± 0.3	nd
α-Cubebene	1,348	nd	nd	nd	nd	0.1 ± 0.0	nd	nd	nd	nd
α-Copaene	1,377	nd	nd	nd	0.1 ± 0.0	nd	0.2 ± 0.0	nd	nd	nd
β-Patchoulene	1,382	nd	nd	nd	nd	nd	nd	nd	nd	5.8 ± 0.5
β-Elemene	1,389	nd	nd	nd	nd	nd	4.8 ± 0.7	3.3 ± 0.1	nd	2.6 ± 0.1
α-Gurjunene	1,410	nd	nd	nd	2.1 ± 0.1	nd	nd	nd	nd	0.1 ± 0.0
β-Caryophyllene	1,419	nd	nd	0.8 ± 0.1	0.3 ± 0.0	2.4 ± 0.3	0.5 ± 0.1	nd	8.2 ± 1.0	6.9 ± 0.5
α-Guaiene	1,430	nd	nd	nd	nd	nd	nd	nd	0.1 ± 0.0	40.6 ± 1.3
α-Bergamotene	1,436	nd	nd	nd	nd	nd	0.4 ± 0.0	nd	nd	nd
Aromadendrene	1,443	nd	nd	nd	1.0 ± 0.1	nd	nd	1.4 ± 0.1	nd	nd
α-Humulene	1,455	nd	1.9 ± 0.3	nd	nd	nd	0.3 ± 0.0	nd	nd	nd
Farnesene	1,457	nd	nd	nd	nd	nd	nd	nd	0.1 ± 0.0	nd
α-Patchoulene	1,460	nd	nd	nd	nd	nd	nd	nd	nd	10.7 ± 0.5
Neoisolongifolene	1,462	nd	nd	nd	nd	nd	nd	nd	nd	1.0 ± 0.1
Alloaromadendrene	1,466	nd	nd	nd	0.7 ± 0.0	nd	nd	nd	nd	4.4 ± 0.2
β-Salinene	1,473	nd	nd	nd	0.3 ± 0.0	nd	nd	nd	nd	nd
*γ*-Muurolene	1,477	nd	nd	nd	0.4 ± 0.1	1.2 ± 0.2	nd	0.3 ± 0.0	nd	nd
Germacrene D	1,485	nd	nd	nd	nd	nd	1.2 ± 0.1	1.4 ± 0.2	nd	nd
Epi-bicyclophellandrene	1,489	nd	nd	nd	0.4 ± 0.1	nd	nd	nd	nd	nd
Aciphyllene	1,492	nd	nd	nd	nd	nd	nd	nd	nd	2.8 ± 0.5
α-Bulnesene	1,498	nd	nd	nd	nd	nd	nd	nd	nd	14.6 ± 0.9
δ-Cadinene	1,507	nd	nd	nd	1.6 ± 0.2	nd	nd	nd	nd	nd
Curcerene	1,511	nd	nd	nd	nd	nd	23.9 ± 2.0	nd	nd	nd
α-Panasinsene	1,519	nd	nd	nd	0.2 ± 0.0	nd	nd	nd	nd	nd
Sesquiphellandrene	1,523	nd	nd	nd	0.1 ± 0.0	nd	nd	nd	nd	nd
*γ*-Cadinene	1,526	nd	nd	nd	nd	nd	0.3 ± 0.0	1.7 ± 0.2	nd	nd
α-Bisabolene	1,539	nd	nd	0.1 ± 0.0	nd	nd	nd	nd	nd	nd
α-Calacorene	1,547	nd	nd	nd	nd	nd	0.8 ± 0.1	nd	nd	nd
***Oxygenated sesquiterpene***										
Methyl eugenol	1,401	nd	nd	nd	nd	0.1 ± 0.0	nd	1.1 ± 0.1	nd	nd
Methyl isoeugenol	1,455	nd	nd	nd	nd	3.1 ± 0.2	nd	nd	nd	nd
Elemol	1,550	nd	nd	nd	nd	nd	nd	3.3 ± 0.5	nd	nd
Spathulenol	1,561	nd	nd	nd	nd	1.7 ± 0.1	nd	nd	nd	nd
Caryophyllene oxide	1,583	nd	nd	0.4 ± 0.0	nd	1.4 ± 0.1	0.4 ± 0.0	nd	0.2 ± 0.0	0.2 ± 0.0
Viridiforol	1,588	nd	nd	nd	0.3 ± 0.1	nd	0.2 ± 0.0	nd	nd	nd
β-Asarone	1,622	nd	nd	nd	nd	85.4 ± 1.1	nd	nd	nd	nd
Cadinol	1,645	nd	nd	nd	nd	nd	0.9 ± 0.1	0.6 ± 0.1	nd	nd
*γ*-Eudesmol	1,625	nd	nd	nd	nd	nd	0.2 ± 0.1	nd	nd	nd
β-Cudesmol	1,649	nd	nd	nd	0.2 ± 0.0	nd	1.1 ± 0.1	nd	nd	nd
Patchoulol	1,668	nd	nd	nd	nd	nd	nd	nd	nd	6.7 ± 0.3
Elemol acetate	1,674	nd	nd	nd	nd	nd	1.2 ± 0.2	nd	nd	nd
α-Bisabolol	1,678	nd	nd	nd	nd	nd	0.2 ± 0.0	nd	nd	nd
α-Asarone	1,679	nd	nd	nd	nd	1.9 ± 0.3	nd	nd	nd	nd
Farnesol	1,706	nd	nd	0.3 ± 0.0	nd	nd	nd	nd	3.0 ± 0.2	nd
Guaiol acetate	1,721	nd	nd	nd	0.1 ± 0.0	nd	nd	nd	nd	nd
Furanoeudesm-1,3-diene	2,091	nd	nd	nd	nd	nd	41.9 ± 2.4	nd	nd	nd
**Others**										
2,6-Dimethyl-5-heptenal		nd	nd	0.2 ± 0.0	nd	nd	nd	nd	Nd	nd
4,8-Dimethyl-3,7-non-adienal		nd	nd	0.1 ± 0.0	nd	nd	nd	nd	Nd	nd

*^*a*^EOs, essential oils; OEO: *Citrus sinensis* (OEO); *Myrtus communis* (MTEO), *Eucalyptus citriodora* (CEO), *Melaleuca alternifolia* (TEO), *Acorus calamus* (AEO), *Commiphora myrrha* (MREO), *Cymbopogon nardus* (CNEO), *Artemisia absinthium* (WEO), and *Pogostemon cablin* (PEO); compounds are listed in order of elution from a HP-5MS capillary column. Identification performed by comparison of mass spectra with the corresponding data in NIST library with respect to total ion chromatogram as well as retention indices, calculated for alkanes C_9_ to C_24_ followed by comparison with the Adams (2007) report. ^*b*^Retention indices on the HP-5MS capillary column. ^*c*^Mean value of three replicates calculated from gas chromatography-mass spectrometry (GC-MS) areas, nd, not detected respective data of NIST and Willey (30: 70) libraries in total ion current (TIC) and the literature, as well as retention indices as calculated according to Kovats (1978), for alkanes C 9 to C 24 compared with those reported by Adams.*

Analysis of volatiles of CEO and CNEO revealed the presence of various terpenes, representing 93.5% and 95.8% of the total oil composition, respectively. These oils were characterized by the presence of predominant acyclic monoterpene aldehyde and citronellal with its respective contents of 81.9 ± 1.1% and 31.5 ± 1,1%, in two oils. Both CEO and CNEO showed higher content of oxygenated compounds, in which the former attributed an appreciably higher content primarily of 91.3% oxygenated monoterpenes. Similarly, CNEO mainly contained oxygenated terpenoids (81.9%) and hydrocarbons (13.9%). Except citronellal, other constituents of CEO were citronellol (5.8 ± 0.7%) and fenchone (2.3 ± 0.3%), while CNEO contained geraniol (30.6 ± 1.3%), citronellol (9.6 ± 0.3%), *l*-limonene (5.7 ± 0.5%), β-elemene (3.3 ± 0.1%), and neryl acetate (2.1 ± 0.2%).

GC-MS analysis of AEO showed identification of 16 mono and sesquiterpenes, accounting for 98.2% of the total oil. Oxygenated terpenes were the major constituents (94.1%) of the oil with the β-asarone being the highest contributor (85.4 ± 1.1%). Methyl isoeugenol (3.1 ± 0.2%), caryophyllene (2.4 ± 0.3%), α-asarone (1.9 ± 0.3%), spathulenol (1.7 ± 0.1%), caryophyllene oxide (1.4 ± 0.1%), and *γ*-muurolene (1.2 ± 0.2%) were also detected. Sesquiterpene content was found relatively higher in AEO (3.7%), whereas monoterpene content was meager (0.4%). Volatile composition of MREO showed abundance of furanoeudesm (41.9%) and curcerene (23.9%), considered as marker components of MREO. Besides these, other sesquiterpenoids such as β-elemene (4.8%), δ-elemene (2.1%), germacrene D (1.2%), elemol acetate (1.2%), and β-cudesmol (1.1%) were also identified.

Total ion chromatogram (TIC) of WEO in GC-MS analysis exhibited characteristic peaks corresponding to 29 mono- and sesquiterpenes, contributing 97.7% of the oil. Oxygenated terpenoids (69.7%) formed the major share of the composition; borneol acetate (26.6 ± 1.7%) and β-terpineol (16.2 ± 0.9%) being the most dominant ones. 1,8-Cineol (10.6 ± 0.5%), linalool (6.4 ± 0.4%), sabinene (5.6 ± 0.3%), *o*-cymene (5.1 ± 0.2%), camphor (3.8 ± 0.5%), and α-pinene (2.7 ± 0.2%) were other important terpenes identified in WEO. Sesquiterpenoids detected in WEO were β-caryophyllene (8.2 ± 1.0%), farnesol (3.0 ± 0.2%), and δ-elemene (2.0 ± 0.3%). Volatile composition of PEO showed various peaks in TIC of GC-MS, representing 15 constituents contributing 97.1% of the oil. Sesquiterpene constituents were highly abundant. Among these, α-guaiene (40.6 ± 1.3%) was the major compound followed by α-bulnesene (14.6 ± 0.9%), α-patchoulene (10.7 ± 0.5%), patchoulol (6.7 ± 0.3%), and β-patchoulene (5.8 ± 0.5%). A comprehensive profile of the chemical composition of the EOs, number of identified compounds, and their group-wise classification is presented in Table 3, which described the number of compounds identified along with their content based on functional groups.

**TABLE 3:** Chemical composition, number of compounds, group-wise classification of EO constituents.

**Classification**	**OEO**	**MTEO**	**CEO**	**TEO**	**AEO**	**MREO**	**CNEO**	**WEO**	**PEO**
Total identified composition (%)	98.9	96.0	93.5	93.3	98.2	83.2	95.8	97.7	97.1
Number of identified compounds	10	9	14	26	16	24	19	29	15
Total monoterpene constituents (%)	98.9	94.1	91.6	85.5	0.9	2.6	82.7	84.1	0.7
Total sesquiterpene constituents (%)	−	1.9	1.6	7.8	97.3	80.6	13.1	13.6	96.4
Total hydrocarbons	98.0	51.5	2.2	50.7	4.1	35.4	13.9	28.0	90.2
Total oxygenated compounds	0.9	44.5	91.0	42.6	94.1	47.8	81.9	69.7	6.9

*EOs, Essential oils; OEO: *Citrus sinensis* (OEO), *Myrtus communis* (MTEO), *Eucalyptus citriodora* (CEO), *Melaleuca alternifolia* (TEO), *Acorus calamus* (AEO), *Commiphora myrrha* (MREO), *Cymbopogon nardus* (CNEO), *Artemisia absinthium* (WEO), and *Pogostemon cablin* (PEO).*

### Nematicidal Activity of Essential Oils

All the test EOs immobilized more than 50% of juveniles of *M. incognita* at different test concentrations. Antinemic activity of the EOs is depicted in Table 4. CNEO exhibited LC_50_ of 325.41, 87.27, and 43.22 μg ml^–1^ concentration after 24, 48, and 72-h exposure, respectively. However, CEO containing a high amount of citronellal showed moderate activity with an LC_50_ of 124.50 μg ml^–1^ after 72 h. OEO rich in *l*-limonene was found to exhibit a comparatively higher nematode toxicity with a lethal concentration LC_50_ of 353.20 μg ml^–1^ within 24 h of J_2_ exposure. The nematicidal activity of OEO enhanced with the exposure time, and the highest activity was recorded at an LC_50_ of 79.35 and 39.37 μg ml^–1^ after 48 and 72 h, respectively (Table 4).

**TABLE 4:** LC_50_ and LC_90_ values (μg ml^–1^) of EOs against *M. incognita*, calculated for three exposure periods in test solutions.

*EOs	**LC_50_ (μ g ml^–1^)			***LC_90_ (μ g ml^–1^)		
	**24 h**	**48 h**	**72 h**	**24 h**	**48 h**	**72 h**
OEO	353.20	79.35	39.37	921.63	556.96	231.70
MTEO	>1,000	932.65	879.40	>1,000	>1,000	>1,000
CEO	746.48	330.41	124.50	>1,000	>1,000	987.42
TEO	404.13	103.64	76.28	>1,000	963.90	943.17
AEO	524.45	90.11	85.23	>1,000	353.21	310.92
MREO	>1,000	>1,000	>1,000	>1,000	>1,000	>1,000
CNEO	325.41	87.27	43.22	912.57	676.28	278.05
WEO	>1,000	937.52	734.72	>1,000	>1,000	>1,000
PEO	>1,000	387.77	290.87	−	>1,000	>1,000

*EOs, essential oils; OEO: *Citrus sinensis* (OEO), *Myrtus communis* (MTEO), *Eucalyptus Citriodora* (CEO), *Melaleuca alternifolia* (TEO), *Acorus calamus* (AEO), *Commiphora myrrha* (MREO), *Cymbopogon nardus* (CNEO), *Artemisia absinthium* (WEO), and *Pogostemon cablin* (PEO). **LC_50_ (μg mL^–1^): Lethal concentration at 50% mortality of nematodes. ***LC_90_ (μg mL^–1^): Lethal concentration at 90% mortality of nematodes.

In this study, TEO and AEO were found effective with an LC_50_ of 76.28 and 85.23 μg ml^–1^ within 72 h, whereas CEO exhibited an LC_50_ of 124.50 μg ml^–1^. MTEO, however, exerted moderate action with an LC_50_ of 879.40 μg ml^–1^. The first three Eos, i.e., OEO, CNEO, and TEO, with an LC_50_ (72 h) below 50 μg ml^–1^ except TEO, were subjected to molecular docking analysis, to understand their possible interaction with proteins for nematicidal action.

### Molecular Docking Study

Seven receptor proteins (putative target proteins) of *M. incognita* were screened against the biomolecules of OEO, CNEO, and TEO, the three most effective EOs in the present study. Gibb’s free energy of binding and other docking parameters of the screened targets are presented in Table 5. Bioactivity of OEO, TEO, and CNEO against *M. incognita* J_2_s was best explained by the *in silico* inhibition of the odorant response gene 1 (ODR1). The binding pocket of the ODR1 allosteric site is composed of 45 amino acid residues. Screening of the compounds present in the three EOs against the ODR1 gave significantly low binding free energy values ranging from -36.9 to 15.3 kcal mol^–1^, suggestive of formation of stable protein–ligand complexes.

**TABLE 5:** *In silico* nematicidal activity of OEO, TEO, and CNEO oil constituents against *M. incognita* ODR1.

**NAME**	**Hbond**	**Hphob**	**VwInt**	**Δ G**	**Heavy_Atoms**	**Log_P**	**LE**	**Relative Percent (>10%)**	**Oil Constituent**
Geraniol	–7.04	–5.05	–18.7	–36.9	11	2.67	0.80	30.6	CNEO
Linalool	–5.08	–5.43	–21.8	–36.2	11	2.67	0.79	<10.0	
Geranial	–2.51	–4.76	–23.7	–33.1	11	2.88	0.72	<10.0	
β-Terpineol	–5.61	–4.95	–15.8	–32.7	11	2.50	0.71	35.7	TEO
*t*-Ocimene	0.00	–5.89	–20.9	–28.9	10	3.48	0.69	<10.0	
Neral	–3.61	–4.76	–21.0	–31.0	11	2.88	0.67	<10.0	
*p*-Menth-8-en-2-ol	–4.95	–5.03	–17.4	–30.4	11	2.36	0.66	<10.0	
Pulegol	–3.80	–5.01	–17.4	–29.9	11	2.50	0.65	<10.0	
2,6-Dimethyl-5-heptenal	–2.58	–4.64	–18.5	–26.8	10	2.57	0.64	<10.0	
Citronellal	–2.66	–4.79	–20.5	–29.3	11	2.96	0.64	31.5	CNEO
Limonene oxide	–1.86	–4.30	–15.6	–28.7	11	2.52	0.62	<10.0	
*l*-Limonene	0.00	–4.74	–15.5	–25.7	10	3.31	0.61	93.2	OEO
β-Myrcene	0.00	–5.42	–21.5	–24.6	10	3.48	0.59	<10.0	
β-pinene	0.00	–4.49	–14.3	–24.2	10	3.00	0.58	<10.0	
β-Pinene	0.00	–4.36	–13.0	–24.1	10	3.00	0.58	<10.0	
β-Terpinolene	0.00	–5.20	–18.5	–24.0	10	3.45	0.57	<10.0	
γ-Terpinene	0.00	–5.58	–17.0	–24.0	10	3.31	0.57	17.5	TEO
Para-cymene	0.00	–5.46	–17.0	–23.9	10	3.12	0.57	<10.0	
4,8-Dimethyl-3,7-non-adienal	–5.24	–5.02	–14.2	–28.6	12	3.27	0.57	<10.0	
Phellandrene	0.00	–5.43	–16.4	–23.6	10	3.16	0.56	<10.0	
α-Terpinene	0.00	–5.18	–18.5	–23.1	10	3.31	0.55	<10.0	
Decanal	–2.45	–5.13	–19.8	–25.4	11	3.33	0.55	<10.0	
Isopulegol	–1.84	–5.04	–18.0	–24.5	11	2.36	0.53	<10.0	
Sabinene	0.00	–5.11	–17.5	–21.0	10	3.00	0.50	<10.0	
δ-Carene	0.00	–5.10	–17.0	–19.8	10	3.00	0.47	<10.0	
Citronellol	–4.66	–5.27	–18.2	–21.6	11	2.75	0.47	9.6	CNEO
Methyl eugenol	0.00	–5.64	–23.9	–25.4	13	2.43	0.47	<10.0	
α-Thujene	0.00	–5.13	–16.3	–19.2	10	3.00	0.46	<10.0	
Neryl acetate	–1.56	–5.92	–21.4	–26.7	14	3.24	0.45	<10.0	
1,8-Cineol	–1.16	–4.88	–14.3	–20.1	11	2.74	0.44	<10.0	
β-Caryophyllene	0.00	–5.72	–17.1	–26.3	15	4.73	0.42	<10.0	
β-Eudesmol	–6.84	–5.95	–8.4	–27.7	16	3.92	0.41	<10.0	
γ*-*Cadinene	0.00	–5.60	–19.5	–25.3	15	4.58	0.40	<10.0	
Epibicyclosesquiphellandrene	0.00	–5.66	–17.8	–25.2	15	4.58	0.40	<10.0	
Sesquiphellandrene	0.00	–7.01	–23.9	–24.2	15	4.89	0.39	<10.0	
Germacrene D	0.00	–5.71	–16.6	–23.9	15	4.89	0.38	<10.0	
α-Panasinsene	0.00	–5.45	–15.3	–23.4	15	4.56	0.37	<10.0	
Cadinol	–5.20	–6.03	–10.5	–24.8	16	3.78	0.37	<10.0	
β-Selinene	0.00	–5.88	–18.5	–22.9	15	4.73	0.37	<10.0	
Allo-aromadendrene	0.00	–5.50	–14.2	–21.2	15	4.27	0.34	<10.0	
β-Elemene	0.00	–6.11	–18.0	–21.1	15	4.75	0.34	<10.0	
γ-Muurolene	0.00	–5.99	–18.2	–20.5	15	4.58	0.33	<10.0	
α-Gurjunene	0.00	–5.43	–15.1	–20.3	15	4.42	0.32	<10.0	
α-Copaene	0.00	–5.91	–16.7	–19.2	15	4.27	0.31	<10.0	
Viridiflorol	0.00	–5.54	–13.7	–20.1	16	3.47	0.30	<10.0	
Guaiol acetate	–1.47	–6.28	–18.5	–23.3	19	4.49	0.29	<10.0	
Elemol	–3.74	–6.28	–12.7	–19.7	16	3.94	0.29	<10.0	
δ-Cadinene	0.00	–6.78	–10.1	–16.3	15	4.73	0.26	<10.0	
Aromadendrene	0.00	–5.79	–11.9	–15.3	15	4.27	0.24	<10.0	

**FIGURE 2:**
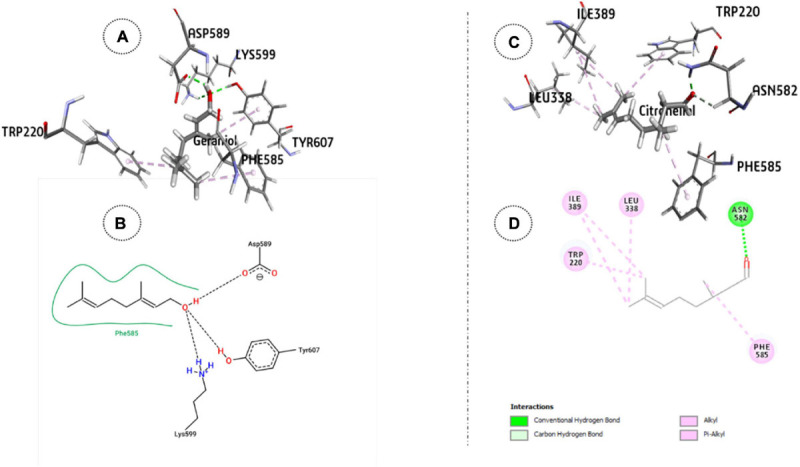
ODR1 bound major CNEO oil constituents: **(A)** 3-D representation of geraniol. **(B)** 2-D representation of geraniol. **(C)** 3-D representation of citronellal and (**D**) citronellal.

The relative stability of the docked complexes of 49 ligands (major compounds, >10% present in the OEO, TEO, and CNEO oils) with the ODR1 was computed in terms of ligand efficiency (Table 5). It can be seen that the lowest binding energy value for the geraniol–ODR1 complex (-36.9 kcal mol^–1^), as depicted in Table 6 may be attributed to the three conventional H-bonds with relatively shorter bond distances (∼2 Å). Additionally, it appeared that the three hydrophobic interactions of π-alkyl type led to further stabilization of the geraniol–ODR1 complex (Table 7). Geraniol was bound specifically to the guanylate cyclase catalytic domain of the ODR1 receptor (Figure 2).

**TABLE 6:** Binding domains in the ODR1 target receptor.

**Domain Name**	**Position (Independent *E*-value)**	**Description**
1 Guanylate_cyc	549……… 724 (2.5e-49)	PF00211, Adenylate and Guanylate cyclase catalytic domain (Adenylate cyclase-activating G protein-coupled receptor signaling pathway and cyclic nucleotide biosynthetic process)
2 PK_Tyr_Ser _Thr	261……… 479 (1.2e-22)	PF07714, Protein tyrosine and serine/threonine kinase (The catalytic domain found in a number of serine/threonine and tyrosine-protein kinases is represented by this entry)
3 HNOBA	498……… 541 (0.0028)	PF07701, Heme NO binding associated (This domain is predicted to function in both bacteria and animals as a heme-dependent sensor for gaseous ligands, and to transduce various downstream signals)

**TABLE 7:** Molecular interaction details of flexible ligand docking of major oil constituents (the top 5) with the ODR1 receptor.

**Constituent**	**Bond between atoms**	**Distance**	**Type of bonding**
Geraniol	:LYS599:HZ2 - Lig:Geraniol:O1	2.24581	Conventional Hydrogen
	:TYR607:HH - Lig:Geraniol:O1	2.07546	Conventional Hydrogen
	Lig:Geraniol:H1 -:ASP589:OD1	2.07777	Conventional Hydrogen
	:TRP220 - Lig:Geraniol:C9	4.53747	Pi-Alkyl Hydrophobic
	:PHE585 - Lig:Geraniol:C10	4.62898	Pi-Alkyl Hydrophobic
	:TYR607 - Lig:Geraniol:C8	5.20422	Pi-Alkyl Hydrophobic
β-Terpineol	Lig:β-Terpineol:H1 -:ASN340:OD1	2.14644	Conventional Hydrogen
	Lig:β-Terpineol:C9 -:ILE389	5.19655	Alkyl Hydrophobic
	:TRP220 - Lig:β-Terpineol	5.04265	Pi-Alkyl Hydrobhobic
	:TRP220 - Lig:β-Terpineol:C9	4.59553	Pi-Alkyl Hydrobhobic
Citronellal	:ASN582:HD22 - Lig:Citronellal:O1	1.86408	Conventional Hydrogen
	:ASN582:HA - Lig:Citronellal:O1	2.5931	Carbon Hydrogen
	Lig:Citronellal:C8 -:LEU338	5.31917	Alkyl Hydrophobic
	Lig:Citronellal:C8 -:ILE389	5.35293	Alkyl Hydrophobic
	Lig:Citronellal:C9 -:ILE389	5.26424	Alkyl Hydrophobic
	:TRP220 - Lig:Citronellal:C9	5.02254	Pi-Alkyl Hydrobhobic
	:PHE585 - Lig:Citronellal:C10	4.31251	Pi-Alkyl Hydrobhobic
*l*-Limonene	:LEU349 - Lig: *l*-Limonene	5.48522	Alkyl Hydrophobic
	:PRO385 - Lig: *l*-Limonene	4.65347	Alkyl Hydrophobic
	Lig: *l*-Limonene:C10 -:VAL383	4.26576	Alkyl Hydrophobic
	Lig: *l*-Limonene:C10 -:PRO385	3.65949	Alkyl Hydrophobic
	Lig: *l*-Limonene:C4 -:LEU349	4.67353	Alkyl Hydrophobic
	Lig: *l*-Limonene:C4 -:VAL383	4.5661	Alkyl Hydrophobic
	:TRP347 - Lig:Limonene	5.44615	Pi-Alkyl Hydrobhobic
γ-Terpinene	:PRO534 - Lig:γ-Terpinene	4.26992	Alkyl Hydrophobic
	:PRO535 - Lig:γ-Terpinene	5.10312	Alkyl Hydrophobic
	:ALA597 - Lig:γ-Terpinene	3.68262	Alkyl Hydrophobic
	:ALA597 - Lig:γ-Terpinene:C8	4.33181	Alkyl Hydrophobic
	:LYS599 - Lig:γ-Terpinene	5.25154	Alkyl Hydrophobic
	:ALA610 - Lig:γ-Terpinene:C9	4.07633	Alkyl Hydrophobic
	Lig:γ-Terpinene:C10 -:PRO534	4.0165	Alkyl Hydrophobic
	Lig:γ-Terpinene:C10 -:VAL613	4.30711	Alkyl Hydrophobic
	Lig:γ-Terpinene:C10 -:PRO614	4.12284	Alkyl Hydrophobic
	Lig:γ-Terpinene:C8 -:PRO535	4.64633	Alkyl Hydrophobic
	:TYR672 - Lig:γ-Terpinene:C10	5.24394	Pi-Alkyl Hydrobhobic

The citronellal–ODR1 complex with two H bonds (one conventional and one C–H type) and five hydrophobic bonds (three alkyl and two π-alkyl types) (Figure 2), exhibited ΔG-29.3 kcal mol^–1^. In this case, the amino acid residues responsible for the ligand binding interactions belonged to both guanylate cyclase and tyrosine/serine/threonine kinase catalytic domains.

**FIGURE 3:**
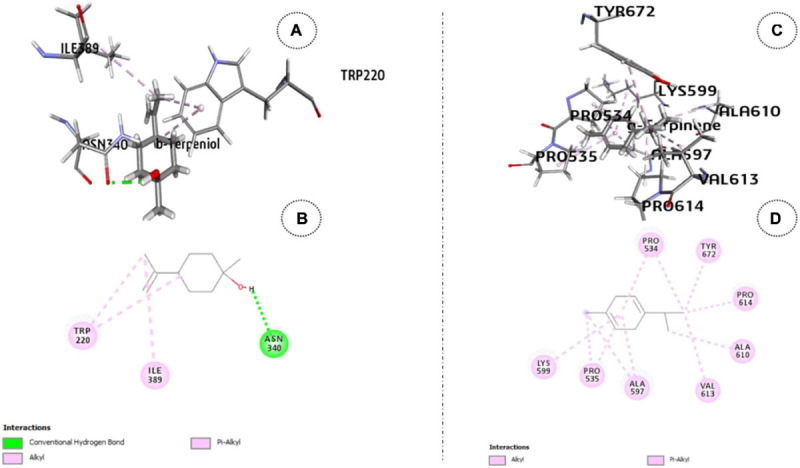
ODR1 bound major TEO oil constituents: **(A)** 3-D representation of β-terpineol. **(B)** 2-D representation of β-terpineol. **(C)** 3-D representation of γ-terpinene and **(D)** 2-D representation of γ-terpinene.

The β-terpineol–ODR1 complex, emerged as the next strongest one with -32.7 kcal mol^–1^ binding energy. One conventional H bond and three hydrophobic bonds (one alkyl type and two π-alkyl types) attributed to complex formation (Figure 3). Here, the specific binding site was tyrosine and serine/threonine kinase catalytic domain.

**FIGURE 4:**
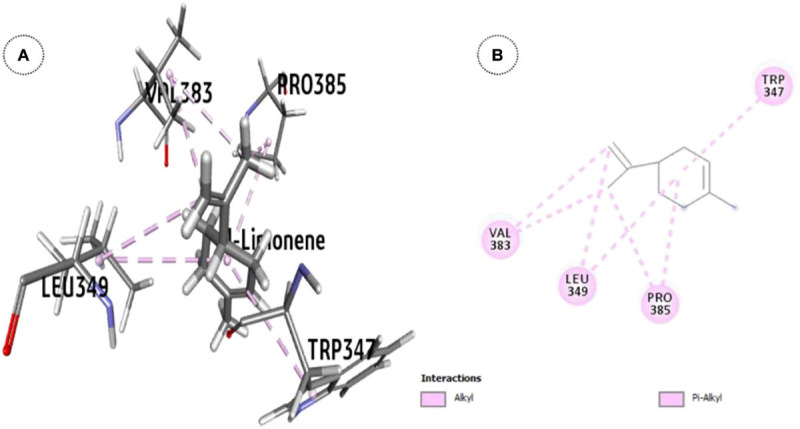
ODR1 bound major OEO oil constituent: **(A)** 3-D representation of *l*-limonene and **(B)** 2-D representation of *l*-limonene.

*l*-Limonene, the major constituent (93.2% w/w) in OEO showed significant inhibition of the ODR1 gene (ΔG = -25.7 kcal mol^–1^). The *l*-limonene–ODR1 complex exhibited seven hydrophobic interactions (six alkyl and one π-alkyl type) in between the protein and the ligand (Figure 4). Apparently, it was bound to the tyrosine/serine/threonine kinase catalytic domain.

The next major EO constituent showing significantly low free energy of binding was γ-terpinene (ΔG = −24 kcal mol^–1^, 17.5% in TEO). The γ-terpinene–ODR1 complex showed 10 hydrophobic interactions (nine alkyl and one π-alkyl type). The γ-terpinene molecule bound to the HNOBA and guanylate cyclase catalytic domains.

Based on the observed relative ΔG values of ligand receptor complexes, *l*-limonene ranked fourth (geraniol–ODR1 complex > β-terpineol–ODR1 complex > citronellal–ODR1 complex > *l*-limonene–ODR1 complex > γ-terpinene–ODR1 complex). Inspite of this, the highest observed *in vitro* nematicidal activity of the OEO oil with *l*-limonene could be possible due to the exceptionally high content of *l*-limonene (93.7%). The major constituents in other active EOs was up to about 36% only (Table 6).

In order to compare the efficiency of smaller ligands with larger ligands in a non-biased manner, ligand efficiency (LE) calculated for the 49 phytochemical constituents of the three oils varied in the range of 0.8–0.24 kcal mol^–1^ HA^–1^. Ninety-one percent of the compounds had an LE above the threshold value of 0.3 kcal mol^–1^ HA^–1^, establishing the discovery of natural leads targeting the ODR1 gene in *M. incognita*. This is the first report on the quantitative binding affinity of the EO constituents toward the ODR1 gene of the root-knot nematode, *M. incognita*, to the best of our information.

## Discussion

In the present study, we performed comprehensive chemo-profiling of EOs in order to understand their possible interactions with the target sites of *M. incognita*. The previously investigated reports on OEO suggested the most prominent monoterpene, *l*-limonene, with a range of 32–98% (Zhang et al., 2019; Matuka et al., 2020). Dejam and Farahmand (2017) described MTEO as primarily composed of monoterpenes such as 1,8-cineol, α-pinene, and linalool, which was further confirmed in our study. However, Tunisian MTEO have been reported to be rich in α-pinene (Jamoussi et al., 2005). In our study, α-pinene has been found to be a major component of MTEO. Bioactive terpenic compositions of CEO make it worthy to study on volatile constituents for diverse biological properties. Contrastingly, the oil contains a high amount of citronellal, citronellol, and isopulegol (Singh et al., 2012; Siddique et al., 2013). An earlier report by Madalosso et al. (2017) and Raymond et al. (2017) described the volatile composition of TEO rich in terpinenes, terpinen-4-ol, and methyl eugenol. Our analysis too revealed that TEO comprised of β-terpineol and terpinene. Methyl eugenol, however, was not detected. Our findings on AEO predominantly containing β-asarone have been corroborated by Deepalakshmi et al. (2016). The present study suggested that industrially important MREO, CNEO, WEO, and PEO contained a higher amount of furanoeudesm 1,3 diene, geraniol, myrcene, camphor, and patchoulol, respectively, as reported previously (Buré and Sellier, 2004; Nguyen et al., 2018; Kalaiselvi et al., 2019). Reported variation in chemical profiles of these EOs could be attributed to the plant sources related to locational, seasonal, and climatic factors.

Plant EOs have been described as having great potential in nematode control (Andrés et al., 2012). Oxygenated monoterpenes particularly aldehydes and alcohols have particularly been found effective against *M. incognita* (Echeverrigaray et al., 2010). A similar trend in activity was demonstrated in the case of CNEO comprising an abundance of citronellal and geraniol (Choi et al., 2007). The activity increased both with increasing concentration of EOs and treatment time. Literature also confirmed that EOs containing higher amounts of *l*-limonene usually showed excellent nematicidal potential (Duschatzky et al., 2004). The relative order of nematicidal activity exhibited by the test EOs after a 72-h incubation period, was OEO > CNEO > TEO > AEO > CEO > PEO > WEO > MTEO > MREO.

## Conclusion

The present study employs analytical and molecular modeling tools to relate the nematicidal activity of potential essential oils and the interactions of their chemical constituents with the target site proteins of the organism. Among the nine essential oils screened against *M. incognita in vitro*, the orange (OEO) and citronella (CNEO) oils were identified in the present work as most effective for immobilization and killing of nematodes. *In silico* analysis suggested a higher binding capacity of geraniol, β-terpineol, citronellal, *l*-limonene, γ-terpinene, to the selected target proteins. Molecular docking-based understanding of the bioactivity of aromatic oils is a novel attempt toward logic-driven selection of natural materials and discovery of biopesticidal leads. The present findings will be further confirmed through wet lab molecular studies and utilized in bionematicide product development.

## Data Availability Statement

The original contributions presented in the study are included in the article/supplementary materials, further inquiries can be directed to the corresponding author/s.

## Author Contributions

AS, AdK, AD, and AM conceptualized the study. AdK, AM, AnK, and SD validated the study. AdK, AD, AM, LN, RP, MM, JA, NP, and PS conducted the investigation. AS provided the resources. AdK, AM, AD, and SM wrote and prepared the original draft. AdK, AM, AD, SS, and RK wrote, reviewed, and edited the manuscript. AS and UR supervised the study. AS acquired the funding. All authors contributed to the article and approved the submitted version.

## Conflict of Interest

The authors declare that the research was conducted in the absence of any commercial or financial relationships that could be construed as a potential conflict of interest.

## Footnotes

1 http://blast.ncbi.nlm.nih.gov

2 https://parasite.wormbase.org//Tools/Blast

3 pubchem.ncbi.nlm.nih.gov
